# Secondhand Smoke Is an Important Modifiable Risk Factor in Sickle Cell Disease: A Review of the Current Literature and Areas for Future Research

**DOI:** 10.3390/ijerph13111131

**Published:** 2016-11-12

**Authors:** S. Christy Sadreameli, Benjamin T. Kopp, Susan E. Creary, Michelle N. Eakin, Sharon McGrath-Morrow, John J. Strouse

**Affiliations:** 1 Eudowood Division of Pediatric Respiratory Sciences, Johns Hopkins School of Medicine, Baltimore, MD 21287, USA; smcgrath@jhmi.edu; 2 Section of Pediatric Pulmonology, Nationwide Children’s Hospital, Columbus, OH 43205, USA; Benjamin.Kopp@nationwidechildrens.org; 3 Center for Microbial Pathogenesis, The Research Institute at Nationwide Children’s Hospital, Columbus, OH 43205, USA; 4 Division of Pediatric Hematology/Oncology/BMT, Nationwide Children’s Hospital, Columbus, OH 43205, USA; Susan.Creary@nationwidechildrens.org; 5 Division of Pulmonary and Critical Care Medicine, Johns Hopkins School of Medicine, Baltimore, MD 21287, USA; meakin1@jhmi.edu; 6 Divisions of Hematology and Pediatric Hematology/Oncology, Duke University School of Medicine, Durham, NC 27710, USA; john.strouse@duke.edu

**Keywords:** secondhand smoke, environmental tobacco smoke, tobacco, sickle cell disease, pulmonary function tests

## Abstract

Sickle cell disease (SCD) is an autosomal recessive hemoglobinopathy that causes significant morbidity and mortality related to chronic hemolytic anemia, vaso-occlusion, and resultant end-organ damage. Tobacco smoke exposure (TSE) through secondhand smoke exposure in people with SCD of all ages and through primary smoking in adolescents and adults is associated with significantly increased morbidity, with increased rates of emergency department visits and hospitalizations for painful vaso-occlusive crises and acute chest syndrome (ACS). Secondhand smoke is also associated with pulmonary function abnormalities in children with SCD who are already at risk for pulmonary function abnormalities on the basis of SCD. TSE is emerging as one of the few modifiable risk factors of SCD. This review discusses the current state of the evidence with respect to TSE and SCD morbidity, discusses potential mechanisms, and highlights current gaps in the evidence and future research directions.

## 1. Introduction

Sickle cell disease (SCD) is an autosomal recessive hemoglobinopathy that causes a severe, life-long illness. Hemoglobin SS (HbSS) is the most common genotype (60%–65% at birth) and usually results in the most severe form of the disease. SCD affects one in 365 children of African ancestry in the United States, one in 16,300 Hispanic children, and smaller numbers in other ethnic groups [1]. There are approximately 100,000 people living in the United States with SCD and over 3 million with sickle cell trait [2]. SCD is also common in people with ancestry from Sub-Saharan Africa, the Middle East, Central and South America, the Caribbean, Greece, India, Italy, and Turkey [3]. The worldwide prevalence of SCD is not known, but it is estimated that over 300,000 infants with SCD are born each year, and many more are carriers (sickle cell trait) [4]. In addition to moderate to severe anemia that typically develops by six months of age, multisystem complications occur as a result of sickled, rigid red blood cells that are prone to hemolysis and lead to vaso-occlusion and resultant tissue damage and systemic inflammation. The clinical symptoms of the disease are typically intermittent, and affected people suffer from acute exacerbations. The most common exacerbations are acute pain and acute chest syndrome (ACS). Chronic pain and chronic organ dysfunction also develop over time. Very few modifiable risk factors have been identified in SCD that are associated with SCD exacerbations (i.e., those that could be targeted to lessen the severity of SCD). These include prophylaxis against influenza (vaccination) and pneumococcal infection (vaccination, penicillin in early childhood) [5], avoidance of exposure to cold water and weather [6], and malaria prophylaxis with bed nets and/or medications in parts of the world where it is endemic [7]. Evidence is mounting that tobacco smoke exposure (TSE), whether first-hand through primary smoking, or secondhand, is a common and important modifiable risk factor for pain [8,9,10], ACS [8,9,10,11,12], and lower airway obstruction [13,14] in children and adults with SCD. 

Smoking is common worldwide, despite ongoing legislation and public health efforts targeted at tobacco avoidance and cessation. Tobacco smoking is the most common cause of preventable death in the world, and smoking prevalence is increasing in many low- and middle-income countries including areas with a high prevalence of SCD; the proportion of the population that uses tobacco is slowly decreasing in many high-income countries [15]. In the United States, tobacco use is the leading cause of preventable death, and cigarette smoke is responsible for 480,000 deaths each year (41,000 of which are due to secondhand smoke exposure) [16]. Globally, tobacco is responsible for 42% of chronic respiratory disease and 10% of cardiovascular disease. The proportion of people with SCD with current TSE worldwide is not known, though the unique risks of this exposure are being increasingly recognized. At present, the National Heart, Blood, and Lung Institute SCD care guidelines recommend interventions (including education and brief counseling) to prevent the initiation of smoking in school-aged children and adolescents [17]. These guidelines also recommend tobacco use screening and counseling for adults with SCD [17]. The guidelines do not mention screening caregivers of pediatric patients with SCD for smoking to identify sources of secondhand smoke exposure.

TSE is associated with significant morbidity in children and adults with SCD, whether through secondhand exposure or through primary smoking [8,9,10,11,12,13]. TSE appears to have negative effects on pulmonary function and pulmonary exacerbations of SCD (i.e., more frequent ACS episodes) [8,9,10,11,13]. It also has a negative impact on other acute complications of SCD, including more frequent painful vaso-occlusive crises [8,9,10]. TSE may exert direct, toxic effects on the airways of people with SCD, may enhance or exacerbate underlying systemic inflammatory processes, may influence pulmonary inflammation, or all of these. Although the dangers of TSE are well known, there has been little research related to TSE in SCD. Given the unique pathophysiology of SCD, it is critical for clinical providers and researchers to understand and consider the role of TSE on SCD progression and management. Therefore, this review will summarize what is known about the prevalence of secondhand smoke exposure and primary smoking in SCD, will summarize what is known about the impact of TSE on SCD pulmonary and systemic complications in SCD, and will highlight some of the areas in which future research is needed.

## 2. Methods

**Figure 1:**
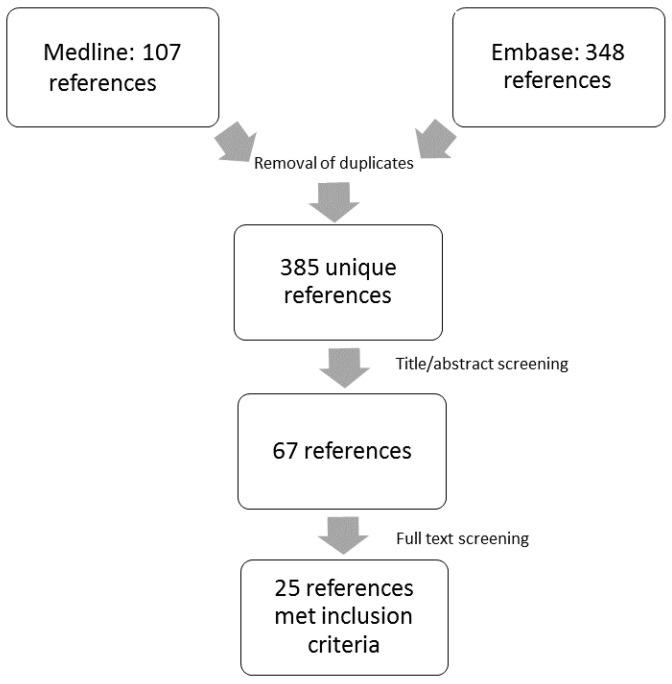
Search and screening results (see Supplementary Materials Figure S1 for search strategy).

We used a systematic search strategy for this narrative review. Search terms (Supplementary Materials Table S1) and search strategy (including Embase and Medline databases) were developed with a clinical information specialist at Johns Hopkins (Baltimore, MD, USA). Our inclusion criteria were: (1) original research publications or published abstracts with (2) SCD (any type) and (3) both of the following: (a) any measurement of tobacco (including cigarette smoking or other tobacco product use, secondhand smoke exposure, or thirdhand smoke exposure); and (b) any reported health or laboratory outcome related to tobacco in SCD. Because animal studies can add important mechanistic insight, we considered human and animal studies eligible. We did not restrict by publication language, geography, or year of publication. We did not restrict based on research methodology, i.e., we included studies that used quantitative, qualitative, and mixed methods. We searched the Embase and Medline databases on 2 August 2016 (Figure 1). A single author performed initial screening of titles and abstracts as well as subsequent full text screening. We did not employ systematic data extraction or a formal analysis tool, and we did not perform quality appraisal of included studies, as published content was limited. Twenty-six publications met criteria for inclusion in our review. We have cited additional publications related to SCD or the health effects of TSE if relevant to the paper.

## 3. Results

### 3.1. Tobacco Smoke Exposure: Sources and Measurement

Several studies have described negative health consequences of TSE in children and adults with SCD. Most pediatric studies have focused on secondhand exposures [9,10,11,13], and only one study has described the prevalence and ill effects of secondhand smoke in adults with SCD [8]. Three adult studies in SCD have focused on the prevalence and/or health consequences of smoking tobacco [8,12,18], and the remainder of the studies of adult SCD patients that we identified included tobacco use as a predictor/variable. There are other sources of exposure to tobacco that are of concern besides combustible cigarettes. These include the use of e-cigarettes, cigars, pipes, chewing tobacco, hookah, and snus. We did not identify any publications related to these types of exposure in SCD. Thirdhand smoke, which is exposure to nicotine and other residual chemicals from a variety of sources (such as furniture, clothes, hair, dust, and the inside of cars) is also a concern and is increasingly being recognized as a cause of morbidity in children and adults [19,20]. However, our search did not reveal any publications in which thirdhand smoke was evaluated in relation to SCD.

The most common method for measurement of TSE in SCD (whether first or secondhand) is indirect: self-report, either by questionnaire or interview. Not surprisingly, direct, biomarker measurements are more sensitive to identify TSE. Compared with biomarkers, parental and self-report may underestimate actual exposure to tobacco [21,22,23]. Under-reporting may be related to the social undesirability of tobacco, and in at least some cases it may be related to unawareness on the part of a parent/caregiver. For example, exposure could occur when a child is in the care of another adult besides the primary caregiver (extended family members, split-parent households, child care arrangements, etc.) and secondhand smoke exposure is particularly common in environments with multi-unit housing [24]. Similarly, adults may be exposed to secondhand smoke in social or occupational settings and may not report consistent exposure to secondhand smoke in the household setting or elsewhere. We identified only one study that included secondhand smoke exposure for adults with SCD [8]. Nicotine and its metabolites can be measured directly in a variety of human specimens. Cotinine is the major metabolite of nicotine and the biomarker of choice for both smoking and involuntary exposure (such as secondhand smoke exposure) [25]. Cotinine has a longer half-life than nicotine (15–19 h compared with nicotine’s 1–2 h). Cotinine may be measured in plasma, saliva, and urine, and values are highly correlated among various bodily fluids [25]. Measurements are quantitative and various cutoffs can be used to identify secondhand exposure and primary smoking. Hair or nail nicotine and cotinine may also be measured and reflect longer periods of exposure. However, added expense and difficulty obtaining specimens are potential downsides to these longer-term forms of measurement. Other direct measurements of TSE include household air nicotine monitors and portable nicotine monitors that attach to a garment. Exhaled carbon monoxide can be used to identify smokers, in whom it is elevated, but is less helpful to identify secondhand exposure. Exhaled carbon monoxide may also be elevated in people with SCD who have high rates of hemolysis, which may interfere with the validity of this technique to identify smokers [26]. Biomarker measurement via biological specimens reflects all sources of tobacco exposure. Plasma, saliva, and urine cotinine are relatively easy and inexpensive to obtain (though salivary cotinine may only be available in the research setting). For research studies in which it is feasible, longer-term direct measurements (i.e., hair and nail measurements) of nicotine and cotinine should be considered, as they reflect a longer period of time and may more reliably identify people who are chronically exposed to tobacco.

### 3.2. Tobacco Smoke Exposure in SCD: Prevalence and Associations

The prevalence of smoking and secondhand smoke exposure among children, adolescents, and adults with SCD worldwide is not known. However, a few recent estimates of secondhand smoke exposure are available from study populations of various sizes. The reported prevalence of secondhand smoke exposure among study populations of children and adolescents with SCD has varied from 27% to 45% (Table 1) [9,10,11,13]. One recent study of American adults with SCD reported that 17% of nonsmokers reported current exposure to secondhand smoke [8]. Estimates of primary smoking have varied considerably based on various study populations. One 1998 study of American adolescents with SCD showed that 30% reported ever smoking, and 6.5% smoked on a regular basis [27]. At the time of the study, this was slightly lower than the rate of smoking among all adolescents in the United States. The rate of smoking among adolescents in the United States has decreased since 1998, and it is possible that smoking among adolescents with SCD has also decreased [27]. A recent questionnaire study among Jamaican adolescents with SCD found that approximately 29% of adolescents had smoked cigarettes, 9% had smoked cigarettes in the last 30 days, and approximately 17% reported having smoked marijuana. Overall, the rates of cigarette and marijuana use were slightly lower in participants with SCD compared with national rates in Jamaica [28]. A recent study showed that the current self-reported smoking rate among American adults with SCD in an unselected cohort was 36% [8]. 

Secondhand smoke exposure has been associated with increased risk for hospital admission and higher hospital costs in SCD compared with children without secondhand smoke exposure in several studies (Table 1) [10] and a similar risk has been noted in nonsmoking adults with SCD who report secondhand smoke exposure (approximately two and a half-fold increased incidence of ACS) [8]. This association has recently been confirmed via measurement of salivary cotinine: children and adolescents with SCD and cotinine ≥0.5 ng/mL, consistent with secondhand smoke exposure, had a greater than threefold higher rate of hospital admission for pain or ACS in an adjusted model compared with those who did not have elevated cotinine (Table 1) [9]. Secondhand smoke exposure was relatively common, with 45% of children with elevated cotinine, in contrast to only 29% with current parental-reported secondhand smoke exposure in a recent multicenter cohort [13]. Physicians were often unaware of the exposure, as physician documentation of secondhand smoke exposure in the medical record identified only 32% of the participants with elevated salivary cotinine [9]. This may in part be due to parental under-reporting of secondhand smoke exposure, which is consistently reported in chronic lung disease disorders [23]. Additionally, current provider screening methods for secondhand smoke exposure may be imprecise, and there may be inconsistent documentation of smoke exposure in the medical record, even when a provider is aware of the exposure.

### 3.3. Tobacco Smoke Exposure: SCD Complications

#### 3.3.1. Painful Vaso-Occlusive Crisis

Acute painful vaso-occlusive crisis is the most common reason for hospitalization among children and adults with SCD [29]. Acute episodes occur when neutrophils and sickled red blood cells block the flow of blood and decrease oxygen delivery to tissues. Pain episodes may occur suddenly, may affect virtually any part of the body, and may be very severe. Severe episodes require treatment with parental opiate medications, and often require hospital admission. Complex interactions between cell endothelium, red blood cells, white blood cells, platelets, and pro-inflammatory components contribute to painful vaso-occlusive crises. Secondhand smoke exposure is associated with increased risk of hospitalization for pain (between twofold and greater than threefold) [8,9,10] (Table 1). Smoking is associated with an increased risk of hospitalization for acute painful vaso-occlusive crisis in adults, but the association between pain and secondhand smoke exposure among nonsmoking adults with SCD was not statistically significant [8]. 

#### 3.3.2. Acute Chest Syndrome

ACS, defined as a new pulmonary infiltrate involving at least one lung segment and accompanied by fever and respiratory signs and symptoms such as oxygen desaturation, chest pain, tachypnea, and/or dyspnea, is the most common acute pulmonary complication in SCD. ACS episodes range in severity and may be life threatening, but even mild episodes require hospitalization. The etiology of ACS is complex and episodes may be multifactorial. Respiratory pathogens, including viruses, encapsulated bacteria, and atypical bacteria may contribute to ACS episodes; infections are particularly common in young children [30]. Bone marrow embolization and pulmonary infarction are also potential etiologies and more common in adults. Secondhand smoke exposure is a significant risk factor for ACS episodes in both children and adults with SCD [8,9,11,13]. Children with SCD and secondhand smoke exposure are seen in the ED 73% more frequently for ACS than unexposed children with SCD (Table 1) [9,10,11,13]. Though the Cooperative Study of Sickle Cell Disease did not show an association between smoking and the risk of ACS [18], two other studies have shown that adults with SCD who are smokers have more frequent ACS episodes [8,12] and one has demonstrated more frequent pain [8] in smokers compared with nonsmokers. Data suggest that secondhand smoke exposure in children and adolescents with SCD may be as harmful as primary smoking with respect to increased risk of ACS and pain [9]. The association between secondhand smoke exposure and ACS in adults was also similar to the risk of ACS among smokers [8]. The mechanisms underlying the association between ACS and TSE are not well understood, but the association is consistent across multiple adult and pediatric studies. TSE increases the risk of respiratory viral infection, and this could partially contribute to the increased risk of ACS associated with TSE, particularly in young children, in whom viral etiologies are especially common. The effects TSE has on the airways and pro-inflammatory and cell adhesion markers may contribute to the increased risk of ACS. The known impairments of secondhand smoke exposure on in-utero and infancy/early childhood lung growth and development [16] may also be a factor in the increased risk of ACS in children with SCD.

#### 3.3.3. Lower Airway Disease: Asthma and Pulmonary Function Abnormalities 

Pulmonary function abnormalities and recurrent wheezing, features that are typically associated with asthma, are common in SCD. There is clinical overlap between some of these clinical features of asthma and SCD, and some may be due to underlying disease processes of SCD. For example, 55%–78% of children and adolescents with SCD are hyperresponsive to methacholine [31,32,33], which is more than twice the prevalence in the general pediatric population (20%–25%) [34,35], and many of the children with SCD with a positive response to methacholine lack other symptoms of asthma (e.g., wheezing), atopic clinical features, or a clinician diagnosis of asthma [32]. Airway hyperresponsiveness has been associated with increased markers of hemolysis, which suggests that some pulmonary manifestations of SCD may be associated with underlying SCD pathophysiology [9]. Therefore, the term “asthma” is an imprecise way to describe the constellation of lower airway abnormalities and pulmonary symptoms in SCD. However, many authors use this term to describe common features of the pulmonary manifestations of SCD, which have some similarities with typical childhood asthma, and a separate term for the syndrome is not currently being used. Between 20% and 48% of children with SCD have a comorbid diagnosis of asthma [36,37,38], and an asthma diagnosis is associated with more frequent pain and ACS compared to children with SCD without a diagnosis of asthma [39]. There is also a two-fold higher risk of mortality in adults with SCD and asthma compared to those with SCD and without asthma [40]. 

The negative effects TSE could have on the lower airways in SCD are concerning, particularly given the well-documented associations between secondhand smoke on childhood asthma and early life lung development [16]; a few authors have examined this at various points in the lifespan among people with SCD. Lower airway obstruction is frequently identified in children and adolescents with SCD (~35%) and has been associated with increased SCD morbidity, even in the absence of a clinical diagnosis of asthma [41,42]. Secondhand smoke exposure is associated with lower airway obstruction in SCD, and children with parental-reported secondhand smoke exposure (current or in infancy) were approximately seven times more likely to have lower airway obstruction (FEV1/FVC ratio below the lower limit of normal) compared with unexposed children with SCD [13]. Another group of authors reported in a published abstract that children with SCD and parental-reported secondhand smoke exposure had an increased (approximately 3-fold higher) odds ratio for abnormal pulmonary function [14]. We found only one study of pulmonary function in SCD that included data about TSE and did not report a negative consequence: a study of 153 West Indian adults with SCD did not show any association between current smoking and FEV1 and FVC [43]. Two studies of adults with SCD from the same authors (Helvaci et al.) with SCD have showed an association between chronic obstructive pulmonary disease (COPD) and smoking status [44,45], which is not surprising given that smoking is a major risk factor for COPD in the general population. The effects of TSE on hemolysis and/or methacholine hyperresponsiveness in SCD have not been described, but secondhand smoke has been associated with a more than two-fold increased likelihood for bronchodilator response in children with SCD (≥12% improvement in FEV1), another measure of lower airway reactivity and inflammation [13]. Though the mechanism(s) of the associations between lower airway obstruction, bronchodilator reversibility, secondhand smoke exposure, and increased SCD severity are unknown, it is plausible that secondhand smoke exposure exacerbates underlying SCD pulmonary injury. TSE may also exert negative effects through other mechanisms related to pulmonary or systemic inflammation. Additionally, objective markers of secondhand smoke exposure and pulmonary function abnormalities are lacking in most studies of SCD to date, which makes finding clear associations difficult. 

#### 3.3.4. Cardiovascular Morbidity

Smoking has been associated with increased cardiovascular risk, including increased risk of stroke, in the general population; however cardiovascular risk associated with TSE in SCD is not well-known. The risk of future, overt cerebral vascular accidents in children with SCD is assessed via transcranial Doppler ultrasound. One small study (a published abstract) of 77 pediatric clinic patients with SCD reported that self-reported secondhand smoke exposure was not associated with elevated TCD velocity [46]. The same authors reported in a separate published abstract that secondhand smoke exposure was associated with a small increase in tricuspid regurgitant jet velocity elevation by echocardiography in children with SCD, but this was not statistically significant [14]. One study of adults with SCD has described an increased risk of stroke among smokers with SCD compared to nonsmokers with SCD [47]. Studies that utilize direct measurement of TSE have not been described, and the stroke risk related to smoking among adults with SCD has not been described in many studies. However, it is concerning that smoking has been associated with stroke risk in adults, who are more likely than children to have SCD-associated pulmonary hypertension or a history of strokes. The risk of stroke among nonsmoking adults with SCD and secondhand smoke exposure has not been described and is also a concern [48]. The association between TSE and other measures of cardiovascular morbidity has not been described. 

#### 3.3.5. Other Effects of Tobacco Smoke Exposure in SCD

Most studies that have related any serious clinical outcomes related to TSE, whether primary or through secondhand exposures, have showed negative health consequences. A published abstract reported an association between risk for central retinal artery occlusion and smoking in adult patients with SCD [49]. One study of adults with SCD confirmed an increased risk of periodontal disease among daily smokers; this effect was similar in magnitude to controls without SCD [48]. Another study showed an association between autosplenectomy and smoking in adults with SCD [50]. Very few studies of people with SCD have showed no effect of TSE, and most of these have been small, with tobacco use included as a covariate/predictor, and not the primary exposure of interest. These include: a retrospective case-control study of adults with SCD in which there were no significant differences in smoking status among those who had experienced a pulmonary embolism and those who had not [51]; a study that examined risk factors for elevated troponin among adults with SCD with acute chest pain that did not find an association between smoking status and elevated troponin [52]; and finally, a study that showed no association between smoking status and decreased bone mineral density in patients with SCD [53]. However, as this was a small study and most patients in the study (80%) had low bone mineral density, this may have limited the authors’ ability to examine the association [53]. This is of questionable clinical significance, as several large studies and meta-analyses have confirmed that smoking is a significant risk factor for low bone mineral density and fractures among the general population [26,54,55]. Two other studies have showed no effect on laboratory parameters related to smoking status in SCD. One of these studies showed that smoking status did not modulate the risk of mutagenicity (measured by mutations in peripheral blood components) related to hydroxyurea use [56]. Another showed that erythropoietin levels were not different according to smoking status in adults with SCD [57]. In summary, the few studies that did not detect a risk associated with TSE in SCD are of questionable clinical significance. Further, TSE may have effects on other end-organ complications of SCD, such as renal disease, but data are lacking. 

#### 3.3.6. Tobacco Smoke Exposure and Mortality

A longitudinal study in Jamaica of 80 subjects with SCD (ages 19–27) examined the associations between cigarette and marijuana smoking, asthma, and pulmonary function in young adults with SCD and compared with race-matched controls without SCD. Current or prior smoking was reported among 21% of the participants with SCD. Smoking was associated with a five and a half-fold increased hazard ratio for death among people with SCD during a 10 year period. Secondhand smoke exposure was not assessed, and participants did not have to specify whether they smoked cigarettes, marijuana, or both [58]. 

#### 3.3.7. Tobacco Smoke Exposure and SCD Morbidity: Potential Mechanisms

In addition to possible direct effects on the airways in SCD, TSE may influence other underlying processes related to SCD severity. For example, TSE, including secondhand smoke, is known to increase pro-inflammatory mediators, up-regulate cellular adhesion factors, damage platelets and endothelium [59,60,61,62], and increase hemolysis of red blood cells [62,63,64,65], all of which contribute to SCD vaso-occlusion and end-organ complications [66]. Hemolysis and free hemoglobin released from lysed red blood cells have also been associated with SCD morbidity and end-organ complications, such as pulmonary hypertension [67]. There are some data to suggest that cigarette smoke introduces oxidative stress and induces alterations of the erythrocyte membrane lipid composition and leads to increased erythrocyte fragility and hemolysis in smoking adults without SCD, [62,63,65] though this has not been studied in people with SCD. A similar effect has been seen in rabbit erythrocytes (i.e., tobacco smoke induced hemolysis) [64]. A single study has examined the role of nicotine on erythrocytes in patients with SCD (*Nicotiana tabacum* leaf extract incubated in vitro with erythrocytes from volunteers with SCD). *N. tabacum* extract caused polymerization of deoxyHbS in a concentration-dependent manner [68]. Though cigarette smoke contains over 4000 chemicals, many of which may have harmful effects on SCD, these data suggest that nicotine itself, which is present in all forms of tobacco products, may increase erythrocyte sickling and exacerbate SCD. Future studies should consider examining the role of hemolysis in SCD morbidity related to TSE. At present, there are no data about the hemolytic rate in people with SCD and secondhand smoke exposure, or data on hemolysis or pulmonary and systemic inflammation in people with SCD who smoke or who are exposed to secondhand smoke. These are areas of potential future study. 

## 4. Areas for Future Research

Despite the risk of SCD morbidity associated with parental and self-reported secondhand smoke exposure, the most recent SCD guidelines do not discuss avoidance of secondhand smoke exposure. This absence in the guidelines highlights the continued knowledge gaps in our understanding of secondhand smoke exposure in SCD and opportunities to apply the existing knowledge on this topic. One major research gap is the lack of longitudinal studies containing objective measures of secondhand smoke exposure and their impact on respiratory function and wheezing in SCD. Decreased forced expiratory volume in one second (FEV_1_) is associated with increased risk of death in SCD [69] and further study of the association between TSE and lung function abnormalities in SCD is essential to understand the role of cessation efforts in preventing SCD respiratory complications. A second gap is the impact of TSE on non-respiratory complications in SCD. TSE, including secondhand smoke exposure, has been shown to impact nutritional status and infectious complications in other systemic disorders such as cystic fibrosis [70], and likely contributes to pain in SCD. TSE may up-regulate underlying inflammatory mechanisms of SCD associated with vascular inflammation and vaso-occlusion, such as cell adhesion, endothelial activation, platelet aggregation, and others [60]. Further, TSE may influence RBC fragility and hemolysis in people with SCD in whom acute and chronic hemolysis is a feature of the disease. TSE is known to have effects on the immune system, and the impact it has on the immune system, if different, in people with SCD is not known. People with SCD have impaired immunity to encapsulated organisms and functional asplenia, and thus the effects of TSE on infection in SCD may be a mechanism of morbidity. Finally, practical applications of secondhand smoke exposure screening and cessation efforts for people with SCD and the caregivers of children with SCD who use tobacco have not been studied. 

## 5. Summary and Clinical Practice Recommendations

Because of the risks associated with TSE for people of all ages with SCD, we recommend that providers screen for and discuss TSE with patients of all ages with SCD (and their caregivers, for pediatric patients). Providers should screen all patients with SCD for secondhand smoke exposure and should counsel patients with SCD and their families about the importance of avoiding secondhand and thirdhand smoke exposure. Considering the underestimation of parental- and self-reported secondhand smoke exposure in SCD and other chronic diseases [9,22,23], routine non-invasive screening measures such as urinary or salivary cotinine or hair nicotine may be useful in addition to subjective screening methods to help guide provider interventions in the clinic and hospital setting. Providers should discourage smoking initiation and proactively screen patients for tobacco use beginning in later childhood to identify those who are most at risk for negative health consequences in order to provide cessation counseling and support. Once TSE has been identified in the clinical setting, providers should encourage methods to limit exposure (such as implementing a home and car smoking ban when secondhand smoke from a household member is present), and to encourage and support cessation for the smoker, whether the smoker is the patient with SCD or another household member. 

## 6. Conclusions

In conclusion, several studies have demonstrated an association between TSE and more frequent hospitalizations and ED visits for pain and acute chest syndrome in SCD [8,9,10,11,12]. Several other studies have suggested other negative impacts related to TSE in SCD, as cited throughout this paper, and one paper has described an increased risk of mortality in adults with SCD who are smokers [58].

Together, existing data combined with knowledge deficits indicate that the effect of TSE on morbidity in SCD is an important area for provider and family education, anticipatory guidance, and additional research. Interventions to increase tobacco cessation and to reduce secondhand and thirdhand smoke exposure are needed to improve the health of children and adults with SCD. Providers should remember to screen patients of all ages with SCD for secondhand smoke exposure and primary use of cigarettes and other tobacco products. Providers should proactively counsel people with SCD, support smokers in their attempts to quit, and provide tools and education about cessation. Finally, researchers should consider including TSE measurements in future clinical studies of people with SCD.

## Figures and Tables

**Table 1:** Risks associated with secondhand smoke exposure in sickle cell disease.

Authors	N Exposed/N Total (% with Secondhand Smoke Exposure)	Population	Exposure Measure	Outcome(s)	Magnitude of Association (95% CI)
West et al., 2003 [10]	22/52 (42%)	Children	Questionnaire	Hospitalizations for pain and ACS (risk ratio)	1.9 (1.3–2.7)
Cohen et al., 2010 [8]	18/106 (17%)	Adults	Questionnaire	Hospitalizations for ACS (risk ratio)	2.62 (1.05–6.57)
Glassberg et al., 2012 [11]	218/810 (27%)	Children	Questionnaire	ED visits for ACS (rate ratio)	1.73 (1.09–2.74)
Cohen et al., 2013 [13]	70/245 (29%) current	Children	Questionnaire	Lower airway obstruction ^†^	22% vs. 3.1% * (*p* < 0.001)
	126/245 (51%) * any (current or prior)			Bronchodilator response >12%	23% vs. 11% * (*p* = 0.03)
Sadreameli et al., 2015 [9]	22/49 (45%)	Children	Salivary cotinine ≥0.5 ng/mL	Hospitalizations for pain and ACS (incidence risk ratio)	3.7 (1.8–8)

* Outcomes pertain to combined current/prior secondhand smoke exposure group; **^†^** FEV1/FVC ratio below the lower limit of normal (below the fifth centile).

## Supplementary Materials

The following are available online at www.mdpi.com/1660-4601/13/11/1131, Table S1: Search strategy.
