# Supplementary material for: Secondhand Smoke Is an Important Modifiable Risk Factor in Sickle Cell Disease: A Review of the Current Literature and Areas for Future Research
Source: Int J Environ Res Public Health. 2016 Nov 12;13(11):1131. doi: 10.3390/ijerph13111131 (PMC5129341; doi:10.3390/ijerph13111131)
Supplement: Supplementary file 1 [file ijerph-13-01131-s001.pdf]

Supplementary Materials: Secondhand Smoke is an Important Modifiable Risk Factor in Sickle Cell Disease: A Review of the Current Literature and Areas for Future Research

S. Christy Sadreameli, Benjamin T. Kopp, Susan E. Creary, Michelle N. Eakin, Sharon McGrath-Morrow and John J. Strouse

Table S1. Search strategy.

**Pubmed**: ("Cotinine"[Mesh] OR "cotinine" OR "Smoking"[Mesh] OR "Tobacco Products"[Mesh] OR "Tobacco"[Mesh] OR "Tobacco Use"[Mesh] OR "Tobacco, Smokeless"[Mesh] OR "Tobacco Smoke Pollution"[Mesh] OR "Nicotine"[Mesh] OR "tobacco" OR "smoking" OR "smoke" OR "nicotine") AND ("Anemia, Sickle Cell"[mh] OR "sickle"[tw] OR "Sickle Cell Anemia"[tw] OR "Sickle Cell anaemia"[tw] OR "Sickle Cell Anemias"[tw]OR "Sickle Cell Anaemias"[tw] OR "Sickling disorder"[tw] OR "Sickle cell disease"[tw] OR "Sickle cell diseases"[tw] OR "Sickle cell disorder"[tw] OR "Sickle cell disorders"[tw] OR "Hemoglobin S disease"[tw] OR "Haemoglobin S disease"[tw] OR "Hemoglobin S diseases"[tw] OR "Haemoglobin S disease"[tw] OR "Drepanocytemia"[tw] OR "Drepanocytic anemia"[tw] OR "Drepanocytosis"[tw] OR "Haemoglobin SS"[tw] OR "Hb SS dieases"[tw] OR "Hemoglobin SS "[tw] OR "Sickle cell anaemia"[tw] OR "Meniscocytosis"[tw] OR "Sickle anaemia"[tw] OR "Sickle anemia"[tw] OR "SS disease"[tw] OR "Hemoglobin SC disease"[mh] OR "Hemoglobin SC disease"[tw] OR "Hemoglobin SC diseases"[tw] OR "Haemoglobin SC disease"[tw] OR "Haemoglobin SC diseases"[tw] OR "Sickle Cell Hemoglobin C disease"[tw] OR "Sickle Cell Haemoglobin C disease"[tw] OR "SC disease"[tw] OR "SC diseases"[tw] OR "Hemoglobin SC"[tw] OR "Haemoglobin SC"[tw] OR "Hb SC disease"[tw] OR "Sickle Cell Hb C disease"[tw] OR "Sickle cell HBC disease"[tw] OR "Sickle haemoglobin C disease"[tw] OR "Sickle hemoglobin C disease"[tw] OR "Hemoglobin SD disease"[tw] OR "Haemoglobin SD disease"[tw] OR "Haemoglobin SD "[tw] OR "Hemoglobin SD"[tw] OR "Hb SD disease"[tw] OR "HbSD disease"[tw] OR "Sickle Cell haemoglobin D disease"[tw] OR "Sickle cell hemoglobin D disease"[tw] OR "Sickle Cell HB D disease"[tw] OR "Sickle Cell"[tw] OR "Drepanocyte"[tw] OR "Meniscocyte"[tw] OR "Sickle Erythrocyte"[tw] OR "Sickler"[tw] OR "Sickle Cell beta thalassemia"[tw] OR "Sickle Cell beta thalassaemia"[tw] OR "Hb S beta thalassemia"[tw] OR "Hb S beta thalassaemia"[tw] OR "Hemoglobin S beta thalassaemia"[tw] OR "Hemoglobin S beta thalassemia"[tw] OR "Haemoglobin S beta thalassaemia"[tw] OR "Haemoglobin S beta thalassemia"[tw] OR "Hemoglobin S thalassemia"[tw] OR "Hemoglobin S thalassaemia"[tw] OR "Haemoglobin S thalassemia"[tw] OR "Haemoglobin S thalassaemia"[tw] OR "Microdrepanocytic anemia"[tw] OR " Microdrepanocytic anaemia"[tw] OR "Microdrepanocytic disease"[tw] OR "Sickle beta thalassaemia"[tw] OR "Sickle beta thalassemia"[tw] OR "Sickle cell thalassemia"[tw] OR "Sickle cell thalassaemia"[tw] OR "Sickle thalassaemia"[tw] OR "Sickle thalassemia"[tw] OR "Thalassemia Hb S disease"[tw] OR "Thalassaemia Hb S disease"[tw] OR "Sickle Cell crisis"[tw] OR "Sickle Cell Trait"[mh] OR "Sickle Cell Trait"[tw] OR "Sickle cell traits"[tw] OR "Sickling trait"[tw] OR "Acute Chest Syndrome"[mh] OR "Acute Chest Syndrome"[tw] OR "Acute Chest Syndromes"[tw])

Embase: ('Cotinine'/exp OR 'cotinine' OR 'Smoking'/exp OR 'Tobacco'/exp OR 'Tobacco Use'/exp OR 'smokeless Tobacco'/exp OR 'passive smoking'/exp OR 'Nicotine'/exp OR 'tobacco' OR 'smoking' OR 'smoke' OR 'nicotine') AND ('Sickle Cell Anemia'/exp OR 'sickle' OR 'Sickle Cell Anemia' OR 'Sickle Cell anaemia' OR 'Sickle Cell Anemias' OR 'Sickle Cell Anaemias' OR 'Sickling disorder' OR 'Sickle cell disease' OR 'Sickle cell diseases' OR 'Sickle cell disorder' OR 'Sickle cell disorders' OR 'Hemoglobin S disease' OR 'Haemoglobin S disease' OR 'Hemoglobin S diseases' OR 'Haemoglobin S disease' OR 'Drepanocytemia' OR 'Drepanocytic anemia' OR 'Drepanocytosis' OR 'Haemoglobin SS' OR 'Hb SS dieases' OR 'Hemoglobin SS' OR 'Meniscocytosis' OR 'Sickle anaemia' OR 'Sickle anemia' OR 'SS disease' OR 'Hemoglobin SC disease'/exp OR 'Hemoglobin SC disease' OR 'Hemoglobin SC diseases' OR 'Haemoglobin SC disease' OR 'Haemoglobin SC diseases' OR 'Sickle Cell Hemoglobin C disease' OR 'Sickle Cell Haemoglobin C disease' OR 'SC disease' OR 'SC diseases' OR 'Hemoglobin SC' OR 'Haemoglobin SC' OR 'Hb SC disease' OR 'Sickle Cell Hb C disease' OR 'Sickle cell HBC disease' OR 'Sickle haemoglobin C disease' OR 'Sickle hemoglobin C disease' OR 'Hemoglobin SD disease'/exp OR 'Hemoglobin SD disease' OR 'Haemoglobin SD disease' OR 'Haemoglobin SD' OR 'Hemoglobin SD' OR 'Hb SD disease' OR 'HbSD disease' OR 'Sickle Cell haemoglobin D disease' OR 'Sickle cell hemoglobin D disease' OR 'Sickle Cell HB D disease' OR 'Sickle Cell' OR 'Drepanocyte' OR 'Meniscocyte' OR 'Sickle Erythrocyte' OR 'Sickler' OR 'Sickle Cell beta thalassemia'/exp OR 'Sickle cell beta thalassemia' OR 'Sickle Cell beta thalassaemia' OR 'Hb S beta thalassemia' OR 'Hb S beta thalassaemia' OR 'Hemoglobin S beta thalassaemia' OR 'Hemoglobin S beta thalassemia' OR 'Haemoglobin S beta thalassaemia' OR 'Haemoglobin S beta thalassemia' OR 'Hemoglobin S thalassemia' OR 'Hemoglobin S thalassaemia' OR 'Haemoglobin S thalassemia' OR 'Haemoglobin S thalassaemia' OR 'Microdrepanocytic anemia' OR ' Microdrepanocytic anaemia' OR 'Microdrepanocytic disease' OR 'Sickle beta thalassaemia' OR 'Sickle beta thalassemia' OR 'Sickle cell thalassemia' OR 'Sickle cell thalassaemia' OR 'Sickle thalassaemia' OR 'Sickle thalassemia' OR 'Thalassemia Hb S disease' OR 'Thalassaemia Hb S disease' OR 'Sickle Cell crisis'/exp OR 'Sickle cell crisis' OR 'Sickle Cell Trait'/exp OR 'Sickle Cell Trait' OR 'Sickle cell traits' OR 'Sickling trait' OR 'Acute Chest Syndrome'/exp OR 'Acute Chest Syndrome' OR 'Acute Chest Syndromes')

© 2016 by the authors. Submitted for possible open access publication under the terms and conditions of the Creative Commons Attribution (CC-BY) license (http://creativecommons.org/licenses/by/4.0/).
